# Pneumaturia

**Published:** 1907-09

**Authors:** 


### SELECTIONS AND ABSTRACTS.

---

### PNEUMATURIA

Taussig says pneumonia may occur either as the result of a rectovesical fistula or of intravesical fermentation. In the latter type the gas may originate either from the fermentaion of glucose in the diabetes, or from the decomposition of proteid substances in cystitis. Of the non-diabetic cases, the microorganism responsible for the pneumaturia has been isolated in only eight cases: in three, it was the bacillus lactis aerognes, and in five the bacterium coli commune (or immobile).

In pneumaturia due to the bacillus lactis aerogenes, the gas formation is readily referable to the peculiar characteristics of the bacillus. In pneumaturia due to the bacterium coli commune, the gas formation may be due (a) to the presence of some proteid not yet definitely determined, that yields a gas when broken down anaerobically by the bacterium coli commune, as suggested by Adrian and Hamm, or (b) to the presence of a peculiar strain of bacterium coli commune that is capable of producing gas where the usual varieties fail to do so.—Boston Medical and Surgical Journal, June 13, 1907.

